# Determinants of neonatal near-miss among neonates delivered in public hospitals of Ilu Abba Bor Zone, Southwest Ethiopia: An unmatched case–control study during the COVID-19 pandemic

**DOI:** 10.3389/fpubh.2022.923408

**Published:** 2022-09-20

**Authors:** Gebiso Roba Debele, Sabit Zenu Siraj, Dereje Tsegaye, Ermiyas Temesgen

**Affiliations:** ^1^Department of Public Health, College of Health Sciences, Mettu University, Mettu, Ethiopia; ^2^Department of Public Health, Mettu Health Science College, Mettu, Ethiopia

**Keywords:** neonatal near miss, determinants, COVID-19, Ilu Abba Bor Zone, Ethiopia

## Abstract

**Background:**

The neonatal period is the time with the highest risk of neonatal and infant mortality. The COVID-19 pandemic diverted resources from routine maternal health services, which raises the possibility of neonatal near misses (NNMs). To implement prompt treatments that could improve the standard of infant care and lower neonatal mortality, it has been theorized that pinpointing the determinants of NNM during this outbreak is crucial. In light of this, the current study identified the determinants of NNM in neonates delivered in public hospitals of Ilu Abba Bor Zone, South West Ethiopia.

**Methods:**

An institution-based unmatched case–control study was conducted among randomly selected 303 (101 cases and 202 controls) neonates admitted to Mettu Karl Comprehensive Specialized Hospital (MKCSH) and Darimu Primary Hospital (DPH) from 1 November to 28 December 2020. Data were collected using interviewer-administered structured questionnaire and checklist. The collected data were coded and entered into Epi-Data version 4.6 and then exported to SPSS version 20 for analysis. Adjusted odds ratios (AOR) along with a 95% confidence interval was used to assess the strength of the association, and a *p*-value < 0.05 was considered to declare the statistical significance in the multivariable logistic regression analysis.

**Result:**

A total of 303 (101 cases and 202 controls) neonates admitted to MKCSH and DPH were included in the study making a 97.4% response rate. In the multivariable logistic regression analysis, no formal maternal education [AOR = 3.534, 95% CI: (1.194–10.455)], Breech presentation during birth [AOR = 3.088, 95% CI: (1.029–9.268)], < 4 antenatal care (ANC) visits [AOR = 1.920, 95% CI: (1.065–3.461], cesarean section delivery [AOR = 4.347, 95% CI: (1.718–10.996)], antepartum hemorrhage (APH) [AOR = 3.37, 95% CI: (1.23–9.24)], and hypertensive disorders of pregnancy (HDP) [AOR = 4.05, 95% CI: (2.36–11.05)] were independent determinants of NNM.

**Conclusion:**

The study's result revealed that factors such as education level, birth presentation, ANC visit, mode of delivery, APH, and HDP continued to be important determinants of the NNM in Ethiopia during this pandemic. Therefore, much work is needed to improve neonatal health by providing adequate ANC services and other identified potential determinant factors that predispose the newborn to life-threatening (near-miss) conditions especially during this pandemic.

## Introduction

The first 28 days of the neonate's lifespan is the principal challenging periodfor the continuity of life for children (1), and it is the most susceptible period that constitutes 50% of neonatal and 75% of infant deaths (2). Although the global mortality rate decreased by 49% from 37 deaths in 1990 to 19 deaths in 2016 per 1,000 live births, a million newborns continued to die in the early neonatal period (3). In Sub-Saharan Africa (SSA), neonatal morbidity and mortality continue to be a large component of the burden of NNM and its rates reflect the efficiency and effectiveness of health care services (4).

Ethiopia was found to be the third highest contributor to neonatal mortality with 187,000 in 2015 (5) and the neonatal mortality rate was 29 per 1,000 live births according to the 2016 Ethiopian Demographic Health Survey (6). Despite a 41% reduction in neonatal mortality in Ethiopia from 49 per 1,000 live births in 2000 to 30 per 1,000 live births in 2019 (7, 8), nearly one out of every ten babies born does not survive to celebrate his/her first birthday (8, 9).

A neonatal near miss (NNM) case refers to an infant who nearly died but survived during birth or within 28 days of extra-uterine life and is being used as a tool to evaluate and improve the quality of care, especially obstetric care (10). In the quest for equitable access and management of quality perinatal care, NNM can be used as a part of the audit system and policymaking together with medical issues (11). Assessing NNM cases provides an all-inclusive predictive factor in neonates delivered from mothers with various obstetric complications (12).

The near-miss approach was used in neonatal health as a revolutionary tool to improve the quality of perinatal care (13), and it provided valuable information to sightsee the quality-of-care issues and set priorities for in-depth healthcare improvements in newborn health (14, 15). Due to its contribution to advancements in the quality of care, healthcare teams are more interested in dealing with morbidity than mortality case reviews (16).

Different studies showed that the NNM rate was 2.6- to 8-fold higher than the neonatal mortality rate (13, 14, 17). The incidence of NNM cases ranged from 21.4 to 85.5 per 1,000 live births (12–14, 17–19). According to the study done in Northwest Ethiopia, the rate of NNM was reported to be 233 per 1,000 live births (20). Although it was planned to end preventable deaths of newborns by 2030 in the Sustainable Development Goal (21), the neonatal morbidity rate still remains high in developing countries (10). To halt this problem, the Ethiopian government has formulated and implemented many policies, including the integrated management of newborn and childhood illness strategy (22), kangaroo mother care (23), and a health sector development plan (24) for the society. Despite these policy and intervention initiatives, currently, Ethiopia has the third highest reported number of newborn deaths in Africa and ranks fifth having the highest number of deaths globally (25).

Due to mild symptoms or remaining asymptomatic and limited testing in developing countries, pregnant women experience an increased risk of maternal morbidity, which increases the likelihood of preterm delivery and admission of their babies to the neonatal unit during the COVID-19 pandemic (26). This maternal and neonatal morbidity is said to be higher among pregnant women in low-income countries (27). In Ethiopia, the outbreak of Coronavirus Disease 2019 (COVID-19) sidetracked resources from routine health services to the control of a pandemic, which has high impact on access to maternal health services (28). All this evidence supports the necessity of assessing the determinants of NNM during the COVID-19 pandemic.

Different studies in SSA countries, including Ethiopia, reported the increment of NNM during the COVID-19 pandemic (29–33). This could be due to the interruption of essential maternal newborn and child health services which has impacted millions of lives across the world (34). Data from various studies indicate that access to routine antenatal, postnatal, and pediatric care is decreasing as a result of the COVID-19 pandemic (35). This indicates that identifying the determinants of NNM is very important and commendable during this pandemic. Thus, the present study aimed to identify determinants of NNM among neonates admitted to selected public hospitals of the public hospitals of Ilu Abba Bor Zone, South West Ethiopia, in 2020.

## Methods and materials

### Study area and study period

The study was conducted in public hospitals of Ilu Abba Bor Zone, which is found in Oromia regional state, South West Ethiopia. The zone is located 554 km far away from Addis Ababa, the capital city of Ethiopia. The MKCSH and DPH are the two public hospitals located in the Ilu Abba Bor area. These hospitals have major clinical departments such as internal medicine, surgery, pediatrics, and gynecology/obstetrics. On average, 56 and 32 neonates were admitted to the neonatal ward of MKCSH and DPH monthly, respectively.

### Study design and period

An institution-based unmatched case-control study was conducted at the MKCSH and DPH from 1 November to 28 December 2020.

### Source population

**Cases:** The source population for the cases were all neonates admitted to the neonatal ward with neonatal near miss cases within 28 days of birth in MKCSH and DPH.

**Control:** The source population for the control were all neonates admitted to the postnatal ward who are free from neonatal near miss cases within 28 days of birth in MKCSH and DPH.

### Study population

**Cases:** Admitted neonates who fulfilled any of the NNM criteria as indicated from their medical records after being identified by a physician within 28 days of birth during the study period in both hospitals.

**Control**: Neonates who are free from any of the NNM criteria as indicated from their medical records after being identified by a physician within 28 days of birth during the study period in both hospitals.

### Inclusion and exclusion criteria

#### Inclusion criteria

**Selecting cases**: All neonates diagnosed with NNM and those who were delivered and admitted to the neonatal ward of both hospitals during the study period were included in the study.

Selection of controls: All neonates with no complication indicated for the selection of cases (free from NNM cases) that were admitted to postnatal care by a pediatrician or a neonatologist or a gynecologist or a resident were enrolled as a control.

#### Exclusion criteria for cases and controls

A neonate with unknown birth history or incomplete medical information was excluded from the study.

### Sample size and sampling procedure

The sample size was estimated using Epi Info-7 software by assuming the confidence level of 95%, power of 80%, and the case-control ratio of 1:2 and by taking the expected percent of exposure in control and odds ratio from previous studies in Ethiopia and presented in Table 1.

**Table 1:** Sample size calculation to identify determinants of NNM among neonates in public hospitals of Ilu Abba Bor Zone, 2020.

**Determinant factor selected**	**% Control exposed**	**Ratio of control to case**	**AOR**	**Power**	**CI**	**Sample size**		**Total**
						**Case**	**Control**	
Distance from a health facility (36)	**44.4%**	**2**	**2.11**	**80**	**95**	**94**	**188**	**282**
Pregnancy type (36)	43.3%	2	2.3	80	95	71	141	212
Birth weight (17)	5.44%	2	4.9	80	95	54	107	161

The final sample size is the maximum of the three, which is 282 (94 cases and 188 control) from the above tables. We added 10% of the non-response rate to both cases and controls and the final sample size was 311 (104 cases and 207 control). The sample was distributed proportionally to the two public hospitals in the zone depending on the number of cases they each handled on a daily basis. Because MKCSH has a monthly case of 56 and DPH has a monthly case of 32, the sample was appropriately distributed using the average total load case. Consequently, the sample size allotted for each hospital was as follows: for MKCSH = 198 (66 cases and 132 controls) and for DPH = 113 (38 cases and 75 controls). Then, the subjects were selected using simple random sampling techniques using registration number as a sampling frame.

### Study variables

Neonatal near miss was the primary outcome of this study and was defined using pragmatic and/or management criteria of the Center Latino-Americano de Perinatology (CLAP**)** (37). Using pragmatic criteria, a neonate with <1,750 g birth weight, a gestational age (GA) of < 33 weeks, and < 7 Appearance, Pulse, Grimace, Activity, and Respiration (APGAR) score at 5 min was used to define NNM. From management criteria, any intubation, parenteral therapeutic antibiotics in the early neonatal period and before 28 days of life; cardiopulmonary resuscitation; phototherapy within the first 24 h of life; the use of anticonvulsants, vasoactive drugs, blood products, surfactants and steroids for hypoglycemia, any surgical procedure, parenteral nutrition, and congenital malformation were used to identify a near miss. A neonate who exhibited at least one of the near miss pragmatic or management criteria but survived this condition within the first 28 days of life was considered a near miss (38). All mothers were followed by a phone call about their child until 28 days after the birth to avoid misclassification of cases and control. Those who died within 28 days of their birth after being classified as a case or control were excluded from the study. As independent variables, we included sociodemographic characteristics, obstetrics-related factors, and maternal- and neonatal-related factors.

### Data collection tool and quality assurance

A pre-tested structured questionnaire, initially prepared in English and translated into Afaan Oromoo, was used to collect the data. The tool contains four parts: sociodemographic-, maternal-, obstetric-, and neonatal-related factors. An interviewer-administered structured questionnaire adapted from relevant literature was used to collect maternal data (10, 13, 16, 36, 38–40). Data on neonate-related factors were collected from medical charts using a standardized checklist and NNM events were collected from neonates' medical records according to the CLAP criteria (37). Two BSc midwives from each hospital and two MPH holders from Mettu Health Science College were recruited for data collection and supervisory duties, respectively. Data collectors were trained for 2 days on the data collection tools and procedures. The data were collected from the postnatal and neonatal wards of both hospitals. Other than this, supervisors were following the data collection process every day during the period of data collection.

### Data processing and analysis

The collected data were coded and checked for their completeness and consistency before data entry. The data were then entered into Epi-Data version 4.6 before being exported to SPSS version 20 for further cleaning and analysis. Data were summarized using the mean with standard deviation for normally distributed continuous variables and the median with interquartile range for non-normally distributed continuous variables, as well as a frequency table with percent for categorical variables. The characteristics of the cases and controls were compared using Pearson's Chi-square test to establish any association between independent variables and the outcome variable. A variable with a *p*-value < 0.2 in a bivariable analysis was a candidate variable for multivariable logistic regression analysis. A sensitivity analysis was conducted to handle the missing data. To evaluate the multicollinearity, a pseudo variance inflation factor (VIF) was used. Then, a multivariable logistic regression analysis was used to identify the presence of the association between dependent and independent variables. The goodness of fitness of the model was checked by the Lemshow–Hosmer test. Statistical significance was determined using 95% confidence intervals of adjusted odds ratios and a *p*-value of < 0.05.

## Results

### Sociodemographic characteristics of mother

A final analysis was done using 303 participants, making a response rate of 97.4%. The mean age (SD) for the cases and the controls was 25.56 (6.386) and 25.94 (5.119), respectively. Less than one-third (23.8%) of cases and 32.2% of controls were found within the age group of 25–29 years. More than two-thirds (71.3%) of mothers in the cases lived in rural areas. In total, the mothers of 39 (38.6%) of cases and 50 (24.8%) of the controls came from at least 5 km away from health facilities. Moreover, the mothers of 91 (90.1%) of cases and 178 (88.1%) of controls were married. In total, the mothers of 43 (43.6%) of the cases and 49 (24.3%) of the controls were housewives (Table 2).

**Table 2:** Socio-demographic characteristics of mothers of neonates admitted (*n* = 303) to public hospitals of Ilu Abba Bor Zone, 2020.

**Variable**	**Category**	**NNM status**		
		**Case (%)**	**Control (%)**	***P*–value**
Age in years	15–19	19 (18.8)	24 (11.9)	0.003
	20–24	25 (24.8)	61 (30.2)	
	25–29	24 (23.8)	65 (32.2)	
	30–34	16 (15.8)	42 (20.8)	
	35^+^	17 (16.8)	10 (5.0)	
Residence	Urban	29 (28.7)	81 (40.1)	0.052
	Rural	72 (71.3)	121 (59.9)	
Distance from health facility	<1 h/5 km	62 (61.4)	152 (75.2)	0.013
	≥1 h/≥5 km	39 (38.6)	50 (24.8)	
Marital status	Never married	4 (4.0)	12 (5.9)	0.002
	Married	91 (90.1)	178 (88.1)	
	Divorced/widowed	6 (5.9)	12 (5.9)	
Educational level	No formal education	20 (19.8)	12 (5.9)	0.002
	Primary	42 (41.6)	84 (41.6)	
	Secondary	30 (29.7)	86 (42.6)	
	More than secondary	9 (8.9)	20 (9.9)	
Occupation	Government	8 (7.9)	26 (12.9)	0.015
	Farmer	25 (25.8)	72 (35.6)	
	Housewife	44 (43.6)	49 (24.3)	
	Merchant	20 (19.8)	42 (20.8)	
	Others*	4 (4.0)	13 (6.4)	

Other*, student, daily laborer.

### Obstetric characteristics of the mothers

In the near-miss group, nearly half (46.5%) of the neonates' mothers were primipara, whereas in the control group more than half (52.0%) of the mothers/caretakers were multipara. More than three-fourths (78.2%) of the fetal presentation during delivery for cases and 184 (91.1%) for the control group were cephalic at birth. Of the neonates' mothers, more than half (59.4%) of the cases and 177 (87.6%) of the control group had mothers with gestational age at birth between 37–41 weeks. In total, mothers of 65 (65.3%) of the neonate cases and 141 (69.8%) of controls had wanted and planned pregnancy. Mothers of nearly two-thirds (65.9%) of the neonates of the cases had less than four antenatal care (ANC) visits, but those of 96 (51.6%) of the controls had > 4 ANC visits during their pregnancy. More than half of the neonates (54, 53%) in cases and (136, 67%) in controls were born *via* spontaneous vaginal delivery (Table 3). In total, 88 (87.1%) of the cases of NNM was due to maternal complications during labor-delivery. Obstruction of labor affected 60.3% of the neonates in cases and 66% of the controls (Table 4).

**Table 3:** Obstetrics characteristics of mothers among neonates admitted (*n* = 303) to public hospitals of Ilu Abba Bor Zone, 2020.

**Variables**	**Category**	**Near miss status**		
		**Case (%)**	**Control (%)**	***P*–value**
Parity	Primipara	47 (46.5)	85 (42.1)	0.012
	Multiparous	39 (38.6)	105 (52.0)	
	Grand multiparous	15 (14.9)	12 (5.9)	
GA at birth	≤ 36 weeks	35 (34.7)	23 (11.4)	0.000
	37–41 weeks	60 (59.4)	177 (87.6)	
	≥42 weeks	6 (5.9)	2 (1.0)	
Fetal presentations during birth	Cephalic	79 (78.2)	184 (91.1)	0.007
	Breech	14 (13.9)	10 (5.0)	
	Transverse/brow/face	8 (7.9)	8 (4.0)	
Current pregnancy type	Wanted planned	66 (65.3)	141 (69.8)	0.418
	Wanted unplanned	18 (17.8)	38 (18.8)	
	Unwanted unplanned	17 (16.8)	23 (11.4)	
ANC during this pregnancy	Yes	88 (87.1)	186 (92.1)	0.167
	No	13 (12.9)	16 (7.9)	
Number of ANC visit	<4 visits	58 (65.9)	90 (48.4)	0.007
	≥4 visits	30 (34.1)	96 (51.6)	
Delivery mode	SVD	54 (53.5)	136 (67.3)	0.001
	C/S	20 (19.8)	13 (6.4)	
	Instrumental	27 (26.7)	53 (26.2)	

Others, oligohydramnios and polyhydramnios; ANC, antenatal care; C/S, cesarean Section; GA, gestational Age; SVD, spontaneous vaginal delivery.

**Table 4:** Maternal complication during labor and delivery among mothers of neonates admitted (*n* = 303) to public hospitals of Ilu Abba Bor Zone, 2020.

**Variables**	**Category**	**Near miss status**		
		**Case (%)**	**Control (%)**	***P*–value**
Maternal Complication during labor and delivery	Yes	88 (87.1)	165 (81.7)	0.167
	No	13 (12.9)	37 (18.3)	
**Type of maternal complication during labor and delivery (among yes)**				
Obstructed labor	Yes	44 (60.3)	66 (66.0)	0.440
	No	29 (39.7)	34 (34.0)	
HDP	Yes	12 (16.4)	8 (8.0)	0.086
	No	61 (83.6)	92 (92.0)	
Hemorrhage	Yes	16 (21.9)	8 (8.0)	0.009
	No	57 (78.1)	92 (92.0)	

HDP, Hypertensive disorders of pregnancy.

### Neonatal-related factors

**Figure 1:**
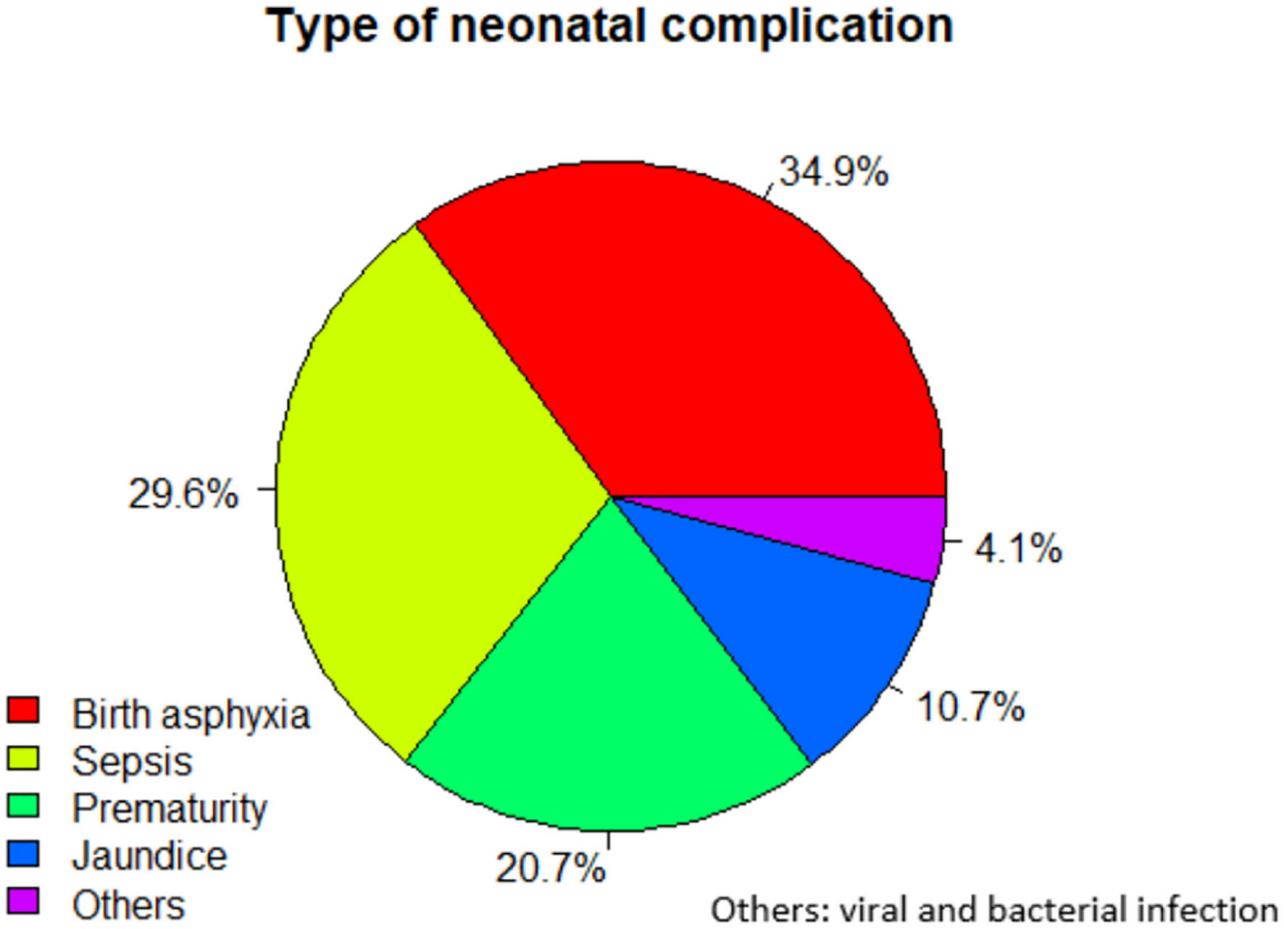
Type of neonatal complications among neonates admitted (*n* = 303) to public hospitals of Ilu Abba Bor Zone, 2020.

More than half (61.4%) of the neonates in cases and 164 (81.2%) of the control group had a normal weight at birth. Two-thirds (67.4%) of the cases had less than an APGAR score at the 5th min of birth (Table 5). An APGAR score of < 7 at the 5th min (22.44%) from pragmatic criteria and the use of intravenous antibiotics up to 7 days and before 28 days of life (26.73%) from management criteria were the most common causes of NNM. Any surgical procedure, congenital malformation, use of corticosteroid for the treatment of refractory hypoglycemia, or use of anticonvulsants, surfactants, and vasoactive drugs were unidentified criteria (Table 6). Birth asphyxia was the most common (34.9%) birth complication of neonates, followed by neonatal sepsis (2936%) (Figure 1).

**Table 5:** Neonatal–related characteristics of NNM among neonates admitted (*n* = 303) to public hospitals of Ilu Abba Bor Zone, 2020.

**Variables**	**Category**	**Near miss status**		
		**Case (%)**	**Control (%)**	***P*–value**
Birth weight of the baby	<2.5 kg	33 (32.7)	30 (14.9)	0.001
	2.5–4 kg	62 (61.4)	164 (81.2)	
	≥4 kg	6 (5.9)	8 (4.0)	
APGAR score at 5th min	<7	68 (67.3)	15 (7.4)	0.000
	≥7	33 (32.7)	187 (92.6)	

**Table 6:** Distribution of NNM conditions among neonates delivered in public hospitals of Ilu Abba Bor Zone, 2020 (*n* = 303).

**Neonatal near miss (NNM) criteria**	**Frequency**	**Percent (%)**
**Pragmatic criteria**		
Gestational age <33 weeks	4	1.32
Birth weight <1,750 g	5	1.65
5th min APGAR score <7	68	22.44
**Management criteria**		
Use of phototherapy in the first 24 h	16	5.28
Cardiopulmonary resuscitation	24	7.92
Any intubation	10	3.30
Transfusion of blood derivatives	1	0.33
Nasal continuous positive airway pressure (NCPAP)	68	22.44
Use of intravenous antibiotics up to 7 days and before 28 days of life	81	26.73

### Determinants of neonatal near miss

The relationship of individual independent variables with the dependent variable was separately analyzed using bivariable analysis. In bivariable analysis, variables such as residence of mother, educational level of the mother, distance from health institution, pregnancy type, parity, fetal presentation, APH, HDP, number of ANC visits, and mode of delivery were significantly candidates for multivariable logistic regression at a *p*-value ≤ 0.2. The variance inflation ranged from 1.42 to 3.20, indicating an absence of multicollinearity among independent variables.

The Hosmer–Lemshow test was insignificant (*p* = 0.576), indicating the satisfaction of the goodness-of-fitness model. In multivariable logistic regression, maternal education, fetal presentation at birth, number of ANC visits, APH, HDP, and mode of delivery were independent significant determinants of NNM. Accordingly, neonates who were born to mothers with no formal education [AOR = 3.534, 95% CI: (1.194–10.455)] and < 4 ANC visits [AOR = 1.920, 95% CI: (1.065–3.461)] had higher odds of experiencing NNM than their counterparts. Neonates with breech presentation [AOR=3.088, 95% CI: (1.029–9.268)], APH [AOR = 3.37, 95% CI: (1.23–9.24)], and HDP [AOR = 4.05, 95% CI: (2.36–11.05)] had a higher risk of NNM. Neonates who were born *via* caesarian section delivery had higher odds of experiencing NNM than neonates born by spontaneous vaginal delivery [AOR = 4.347, 95% CI: (1.718–10.996)] (Table 7).

**Table 7:** Determinants of NNM among neonates admitted (*n* = 303) to public hospitals of Ilu Abba Bor Zone, 2020.

**Variables**	**Neonatal near miss**		**COR (95% CI)**	**AOR (95% CI)**
	**Case N^°^**	**Control N^°^**		
**Residence of mother**				
Urban	29	81	1.00	1.00
Rural	72	121	1.66 (0.99–2.78)	1.04 (0.54–1.99)
**Distance from health facility**				
<1 h/5 km	62	152	1.00	1.00
≥1 h/≥5 km	39	50	1.91 (1.15–3.19)	1.07 (0.54–2.13)
**Educational level of mother**				
No formal education	20	12	3.70 (1.28–10.73)	3.53 (1.19–10.45)^***^
Primary	42	84	1.11 (.47–2.65)	1.81 (0.91–3.60)
Secondary	30	86	0.78 (.318–1.89)	0.28 (0.07–1.11)
More than secondary	9	20	1.00	1.00
**Parity**				
Primipara	47	85	1.00	1.00
Multipara	39	105	0.44 (.191–1.02)	0.60 (0.33–1.10)
Grand multipara	15	12	0.30 (0.13–0.69)	0.52 (0.14–1.90)
**Fetal presentation during birth**				
Cephalic	79	184	1.00	1.00
Breech	14	10	3.26 (1.39–7.65)	3.09 (1.03–9.27)^**^
Transverse/brow/face	8	8	2.33 (0.84–6.43)	1.28 (0.36–4.54)
**Current pregnancy type**				
Wanted planned	66	141	1.00	1.00
Wanted unplanned	18	38	1.01 (.54–1.90)	0.961 (0.445–2.073)
Unwanted unplanned	17	23	1.579 (.79–3.15)	1.158 (0.455–2.952)
**Number of ANC visit**				
<4 visits	58	90	2.06 (1.22–3.49)	**1.92 (1.07–3.46)** ^***^
≥4 visits	30	96	1.00	1.00
**Mode of delivery**				
SVD	54	136	1.00	1.00
C/S	20	13	3.87 (1.80–8.34)	**4.35 (1.72–10.99)** ^**^
Instrumental	27	53	1.28 (0.73–2.25)	1.01 (0.52–1.97)
**APH**				
No	194	85	1.00	1.00
Yes	8	16	4.56 (1.88–11.07)	3.37 (1.23–9.24)^***^
**HDP**				
No	194	89	1.00	1.00
Yes	8	12	3.27 (1.29–8.28)	4.05 (2.36–11.05)^***^

** Indicates p–value of ≤ 0.01 and

*** p–value of < 0.001; ANC, antenatal care; APH, antepartum hemorrhage; C/S, cesarean Section; HDP, hypertensive disorder of pregnancy; SVD, spontaneous vaginal delivery.

## Discussion

Identifying the determinants of NNM in SSA countries such as Ethiopia is very important to decrease neonatal mortality during this pandemic period. Therefore, the current study disclosed the determinants of NNM cases in Ethiopia. Of the characteristics that were assessed, maternal education, number of ANC visits, fetal presentation, mode of delivery, APH, and HDP were found to be determinants of the NNM.

Cases whose mothers had no formal education had higher odds of experiencing NNM than controls. This is consistent with studies done in northeast Ethiopia (41), Ghana (42), and India (13), which showed the significant effect of having an education on NNM. The reason might be that mothers who had no formal education have low or delayed health-seeking behavior and fail to utilize appropriate medical/health care services compared with educated mothers, but these factors may vary from country to country.

Fetal presentation during birth was another factor significantly associated with NNM cases. Neonates with the breech presentation were more likely to have NNM compared to those with the cephalic presentation. This finding is congruent to evidence from southern Ethiopia (16), Southwest Ethiopia (43), and Australia (44). Those studies found that neonates who had a non-vertex presentation were more likely to become near miss as compared to vertex presentation. This might be due to a high risk of birth asphyxia, trauma, and other complications caused by malpresentation during pregnancy (45). Malpresentation may lead to obstructed and prolonged labor, which can result in NNM through different complications to the newborn (45).

In the present study, NNM was higher among neonates whose mothers had < 4 ANC visits, which was supported by studies in Ambo University Referral Hospital and Ambo General Hospital (36). This finding indicates that health facilities need to continue encouraging mothers to receive more frequent ANC visits, which is also emphasized in the WHO recommendation on ANC (46). This finding is also supported by the findings in India (10) and Brazil (38, 47), which revealed higher odds of NNM events among pregnant mothers who had taken less than the minimum required ANC visits during their pregnancy. This could be due to the pregnant woman avoiding preventable risk factors after receiving ANC, through early identification, treatment, and screening for issues that occurred during pregnancy (16). The other possible reason for this finding could be the impact of the COVID-19 pandemic on ANC follow-up. Due to fear of contracting the virus and over-stretched health systems with disrupted supply chains, ANC follow-up is being decreased and below the World Health Organization recommendations, especially in developing countries including Ethiopia (35). However, other studies in Brazil (41) and Morocco (42) revealed insignificant associations between NNM and ANC follow-up. The reason for this difference might be due to varieties in study population and difference in coverage of ANC in different countries.

The current study shows that neonates who were born by cesarean delivery had higher odds of experiencing NNM than neonates born by spontaneous vaginal delivery. Similar studies done in Ethiopia revealed that neonates who were born at the government hospital and health institutions by cesarean section had a higher risk of NNM (48). In line with this study, evidence from Brazil indicated that cesarean section delivery increases the likelihood of experiencing NNM than vaginal delivery (42, 49). This might be due to an increased risk of low 5th min APGAR score, preterm birth, and neonatal resuscitation by cesarean delivery, all of which jointly predispose NNM (50, 51). In other words, as a result of fear of contracting COVID-19, most pregnant mothers are preferring to deliver at home, which may lead to maternal and neonatal complications, including prolonged labor. These impacts might increase the number of cesarean section deliveries.

Hypertensive disease of pregnancy increased the odds of NNM by four times as compared to those mothers who had no HDP.

This finding was similar to the study conducted in Brazil (19, 52), Suriname South America (53), and Ethiopia (43, 54, 55). The study conducted in 29 low- and middle-income countries reported that HDP causes 9% of fresh late fetal deaths and 10% of early neonatal deaths (56). In Ethiopia, HDP account for approximately 7% of perinatal mortality, which may be responsible for the highest perinatal mortality rate in SSA (57). This could be due to the disturbance of vascular manifestations, oxidative stress, and endothelial damage that results from HDP (58). These effects may result in poorer perfusion and nutrient supplementation to the fetus, which enhances adverse perinatal outcomes (58). The possible reason might be that HDP may cause intrauterine fetal complications such as intrauterine growth restriction and preterm delivery and also causes birth asphyxia (59).

The odds of facing NNM were four times higher among neonates born to mothers who had APH in the recent pregnancy than those who had no APH. In congruent with the current finding, studies done in Ethiopia (16) and Zimbabwe (60) found a significant positive effect of APH on NNM. The possible reason could be because bleeding causes oxygen inadequacy for fetal circulation in the uterine, which in turn leads to neonatal morbidities (61, 62).

The current study's findings were intended to provide health professionals with information about factors that determine NNM so that they might take action to minimize risk and increase prevention efforts. In addition, the findings of this study are significant for improving public health since they will help to minimize the financial loss caused by this problem.

Despite its strength, this study was not done without limitations. This study did not incorporate some of the variables that are addressed in the community, such as wealth index and nutritional status.

### Conclusions and recommendations

This study found that the lack of formal education, non-vertex presentation during birth, < 4 ANC visits, cesarean section delivery, APH, and HDP are all significant determinants of NNM. Current findings may provide information that can contribute to the global neonatal and maternal morbidity research agenda about the most frequent complications related to the NNM. In line with our findings, more attention is needed during delivery by healthcare providers, and they need to strengthen and advise all pregnant women for ANC follow-up. Furthermore, targeted ANC follow-up of women is required for a practical approach to reduce NNM by helping at-risk mothers plan for delivery.

## Data availability statement

The raw data supporting the conclusions of this article will be made available by the authors, without undue reservation.

## Ethics statement

Ethical clearance and approval letter to conduct the study was obtained from Research and Ethical Review Committee of Mettu University, College of Health Science. The patients/participants provided their written informed consent to participate in this study.

## Author contributions

All authors equally contributed to the conception, design of the study, acquisition of data, supervision of data collection, analysis and interpretation, and drafting or revising of the article. They have agreed on the journal to which the article will be submitted, gave final approval of the version to be published, and agreed to be accountable for all aspects of the work.

## Funding

Mettu University has covered the costs of data collectors and supervisors per diem. However, the University had no role in the study design, data collection and analysis, the decision to publish, and the preparation of the manuscript.

## Conflict of interest

The authors declare that the research was conducted in the absence of any commercial or financial relationships that could be construed as a potential conflict of interest.

## Publisher's note

All claims expressed in this article are solely those of the authors and do not necessarily represent those of their affiliated organizations, or those of the publisher, the editors and the reviewers. Any product that may be evaluated in this article, or claim that may be made by its manufacturer, is not guaranteed or endorsed by the publisher.
